# Selective Influence of Smoking on Periodontal Treatment Outcomes after 3 Years of Follow-up

**DOI:** 10.3290/j.ohpd.a45087

**Published:** 2020-09-04

**Authors:** Taoufik Boulaamaim, Henri Tenenbaum, Jean-Luc Davideau, Olivier Huck

**Affiliations:** a Postgraduate in Periodontology, University of Strasbourg; Private practice in Rabat, Morocco. Collected the data, wrote the first draft of the manuscript.; b Professor Emeritus, Department of Periodontology, Dental Faculty, University of Strasbourg, Strasbourg, France. Study concept, edited and commented on the manuscript.; c Full Professor, Department of Periodontology, Dental Faculty, University of Strasbourg, Strasbourg, France. Study concept, analysed the clinical data, wrote the final draft.; d Full Professor, Department of Periodontology, Dental Faculty, University of Strasbourg, Strasbourg, France. Study concept, analysed the clinical data, edited and commented on the manuscript.

**Keywords:** nicotine dependence, periodontitis, smoking, treatment outcomes

## Abstract

**Purpose:**

The impact of smoking habits on periodontal treatment has not been clearly elucidated. This study aimed to specify the effects of cigarette consumption and nicotine addiction on periodontal therapy.

**Materials and Methods:**

In this retrospective case-control study, 20 moderate smokers and 20 non-smokers with severe periodontitis were examined after initial diagnosis, and non-surgical active and supportive therapies for 1-6 years (mean follow-up = 3.37 years). Fagerström’s test of nicotine dependence (FTND) was evaluated at re-examination. Treatment efficacy was assessed by periodontal pocket probing depth (PPD) changes and number of teeth lost per year (TL). Bayesian multilevel and regression analyses were performed at site, tooth, and patient levels.

**Results:**

During the mean follow-up period of > 3 years including active and supportive periodontal therapies, mean PPD, PPD > 3 mm and PPD > 7 mm percentage reductions were 1.03, 1.48 and 2.57 times statistically significantly less pronounced, respectively, in smokers than in non-smokers. Multilevel analysis showed that the variability of PPD > 7 mm reduction was mainly associated with patient-level factors. Smokers presented a higher risk for periodontitis progression. In smokers, periodontal parameter improvement was less pronounced in the maxilla and molars. The mean TL was related to the FTND score, not to cigarette consumption. Regression analysis did not demonstrate other influences of demographic and periodontal treatment characteristics on treatment outcomes, except patient age.

**Conclusion:**

Smoking negatively impacted periodontal treatment outcomes at specific tooth sites (deep pockets, maxillary molars) and periodontitis progression, independent of other risk factors.

Cigarette smoking is a well-established risk factor for periodontitis.^41^ Numerous studies have shown that smoking can also negatively impact short- (<12 months),^14,15,59^ medium- (between 1 and 5 years),^9,13,20,42,63^ and long-term (>5 years) periodontal treatment outcomes.^5,12,17,30,36,37,54,61^ Furthermore, a recent meta-analysis demonstrated that smoking cessation significantly improved periodontal treatment outcomes,^34^ suggesting that smoking cessation intervention was a relevant element of periodontal therapy in smokers.^7,41^ However, regarding the relatively low efficacy of tobacco cessation services delivered in dental clinics – resulting in under 6% prolonged abstinence^21^ – practitioners must still consider the therapeutic approach for non-quitting smokers.^47^

This therapeutic approach should be based on a reliable evaluation of smoking risk.^7^ However, smoking risk characteristics and impact on periodontal treatments have not been clearly established, especially for non-surgical treatments.^10,24,31^ Indeed, some studies found no evidence of smoking affecting various treatment outcomes, including mean periodontal pocket depth (PPD) reduction,^2,8,18,27,43,45,49,56,63^ bone level,^43^ tooth loss,^37,51^ or periodontitis progression.^19^ Consequently, it could be difficult for the clinician to evaluate the real impact of patient smoking habits on treatment efficacy.^9^ These apparent discrepancies between studies may be related in part to the variability of the phases of periodontal treatment investigated, as well as data analysis.^9,24,31,63^ In fact, most of the studies only considered initial active periodontal therapy (APT) in the very short term – no more than 3 months^15,18,27,56,59^ – and supportive periodontal therapy (SPT),^5,19,37,42^ which did not reflect the overall effect of smoking on periodontal treatment. Furthermore, smoking has a greater influence on PPD changes during treatment than on clinical attachment loss (CAL).^9,15,31,48,63^ In some cases, only mean PPD changes of initial diseased sites (PPD > 4 mm) were impacted by smoking.^63^ Multilevel analysis, which considers the specific influence of tooth and site on periodontal treatment outcomes, allows a more accurate analysis of smoking effects than a single/patient-level analysis based on a mean effect.^63^ The problem of hierarchical structure of the periodontal response after treatment is prevalent in periodontal epidemiologic research.^62^ This problem will become even more acute in the near future, with the increasing availability of large, complex data sets. Recently, the Bayesian multilevel approach has been proposed to augment traditional analysis in dental and periodontal research.^39,53^ For instance, this statistical model provides a more accurate determination of site subgroups that influence examiner agreement for periodontal measures.^53^ This new approach could be of interest to evaluate the exact probability of smoking effect per patient from the given data and allow data analysis with small groups.^55^

The quantity and duration of tobacco consumption greatly influences the morbidity of smoking.^37,41^ Additionally, other behavioural and psychological aspects associated with smoking, including an increase of stress, could also influence periodontal responses.^4^ The Fagerström test of nicotine dependence (FTND) was initially established to predict smoking-cessation programme efficacy.^35^ FTND is a standard instrument to evaluate the intensity of physical addiction to nicotine. It contains six items that evaluate the quantity of cigarette consumption, the compulsion to use and the dependence. FTND does not only reflect a quantitative aspect of smoking but is also independently correlated with patient behaviour characteristics,^40^ systemic responses^52^ and the risk of cancer.^32^

We hypothesise that smoking and nicotine addiction have a differential effect on periodontal treatment outcomes according to the type of lesion, tooth involved and periodontitis progression. In order to evaluate the smoking and nicotine addiction impact on outcomes of overall periodontal treatment including APT and SPT in current moderate smokers versus non-smokers with severe periodontitis, a retrospective case-control study using a Bayesian multilevel analysis was performed.

## Materials and Methods

### Patients

Patients attending regular SPT visits at the Department of Periodontology, Dental Faculty, University of Strasbourg, France, were examined from January 2009 to June 2010. The present study was conducted following the ethical principles of the Declaration of Helsinki (2008 version); Strasbourg’s Hospital Ethics Committee granted ethical approval (ref: 2015-78). Records and charts of patients seen for an initial examination from 2002 to 2009 were selected and patients were invited to participate in this study. Patients were divided into two groups (smokers and non-smokers).

### Sample Size Calculation

The sample size was based on the mean PPD reduction and standard deviation at the patient level observed in comparable studies^14,30,63^ and computed using G*power 3.1 software (University of Düsseldorf, Düsseldorf, Germany) as follows: the expected difference of mean PPD reduction between smokers and non-smokers was 0.3 mm and the standard deviation was 0.35 mm. Forty patients were selected, with 20 patients in each group.

### Initial Examination Records and Exclusion Criteria

For the patient to be included in the study, the medical and periodontal history, PPD, number of missing teeth, and smoking status had to be recorded. PPD was measured using a manual periodontal probe PCPUNC 15 (HuFriedy; Chicago, IL, USA) at six sites per tooth. Patients who had undergone periodontal treatment before the initial examination, except for supra-gingival scaling, were excluded. Patients were categorised as severe chronic/aggressive periodontitis according to the classification of AAP^3^ (corresponding to periodontitis stages 3 to 4, grades A to C^44^ and presenting at least 10% of sites with CAL ≥ 5 mm). Aggressive periodontitis was defined by rapid attachment loss, bone destruction, and local (amount of microbial deposits) and systemic risk factors inadequate for periodontal destruction.^33^ Patients who used medications such as calcium channel blockers, were in need antibiotic prophylaxis, suffered from diseases that could influence periodontal status and treatment such as diabetes, and had ≤ 10 teeth (excluding third molars) were not included.

### Nonsurgical Initial Periodontal Therapy

After initial diagnosis, non-surgical APT was performed, including oral hygiene education, scaling and two or three sessions of root planing. Oral hygiene was controlled at each visit and hygiene instructions were repeated if needed. Anti-infective systemic therapy was additionally given to patients with marked periodontal tissue inflammation and/or aggressive periodontitis,^22^ consisting of a course of systemic metronidazole (250 mg) and spiramycin (1,500,000 IU) twice a day for 15 days.^33^

### Supportive Periodontal Therapy

The criteria to enroll patients in SPT were a significant and stable reduction of plaque accumulation, gingival inflammation and number of sites ≥ 4 mm. At the end of APT or at SPT re-evaluations, the recommended interval was 3-4 months for patients having residual PPD ≥ 5 mm associated with BOP and/or more than 10% of PPD ≥ 4 mm and/or 10% of BOP. For other patients, a 6-months interval was recommended. Each SPT session included a complete periodontal examination and, if needed, a reinforcement of oral hygiene methods. Subgingival biofilm and calculus (if needed) was carefully remove in residual and recurrent PPD ≥ 4 mm. Periodontal surgery (open-flap debridement) was performed during SPT in case of persistence (at least 6 months after APT completion) or worsening of deep periodontal defects (PPD > 5 mm associated with multi-rooted teeth).^33^ APT/SPT examinations and treatments were performed by trained periodontists or by students under supervised by experienced periodontists (HT, JLD, OH) at the Department of Periodontology.

### Clinical Re-examination

All re-examinations were performed by the same examiner (TB) at the Department of Periodontology from January 2009 to June 2010. Patients were informed about the aims of this study and provided their consent orally. Re-examination included a medical history and self-reported comprehensive smoking history. Patients were categorised as smokers or non-smokers. The minimum consumption of smokers was 5 cigarettes/day without cessation during the follow-up period.^12^ Non-smokers were those who never smoked in their lives.^5^ For smokers, nicotine dependence was evaluated using FTND.^25^ A family history of periodontal disease and previous periodontal treatment modalities were recorded. The number of teeth lost/extracted during the study period was noted. The primary criterion of evaluation was mean PPD changes. The secondary criteria were % of PPD > 3 mm and % PPD > 7 mm changes and tooth loss.

### Bayesian Data Analysis

The percentage of compliance was estimated by dividing the number of visits attended by the number of initially scheduled visits. The number of teeth lost during the follow-up period was divided by the number of follow-up years (TL). Worsening sites were defined as a PPD increase > 2 mm^19^ between the initial and final examinations. Periodontitis progression was defined as at least 3 sites on two different teeth with PPD increase > 2 mm, as adapted from Tonetti and Claffey^60^ (at least 3 sites on two different teeth with CAL increase > 2 mm). Third molars were excluded from the analysis.^20,63^

The influence of smoking on the treatment outcomes of periodontitis was evaluated using multilevel multiple regression. In order to consider correlation between sites, teeth, and patients, three levels were considered in each model with random effects, under the assumption of normal distribution. To compare mean PPD before and after treatment in each group, the multilevel model was fitted under a gamma distribution assumption. To study proportions of sites where PPD was > 3 (total number of pockets) or 7 mm (deep pockets) and the number of worsening sites, multilevel logistic regressions with binomial distribution were fitted to the data. To compare changes of periodontal parameters on molars or on archs, interactions from both the second and third order were included in the model. Models results are described as mean ± standard deviation and 95% credibility interval or as percentage for the coefficient to be positive. Statistical analyses were run under the Bayesian paradigm.^6,38,39,55^ The Bayesian paradigm gives the probability that a treatment or that an effect is present, given the observed data, while the frequentist methods give the probability of the data given a null hypothesis. The results are expressed as point estimates (mean, proportion or difference thereof, or correlation coefficient) and a 95% credibility interval. Finally, Bayesian analysis results provide the probability that the parameter under study is larger (or smaller) than a reference value. Very high (>0.95) or very low values (<0.05) of this probability (pr) can be considered statistically significant. Computations were run with R 3.0.2 and WinBUGS 1.4 (MRC Biostatistics Unit; Cambridge, UK). For each analysis, a single MCMC (Markov Chain Monte Carlo) chain with 5000 iterations as burn-in and 100,000 iterations were used to generate the posterior distribution. Autocorrelation and convergence were checked graphically. The model converged in every case.

## Results

### Population Characteristics at Initial Examination and Treatment Modalities

At the initial examination, the demographic characteristics were comparable for the two groups. The mean number of cigarettes/day (cig/day) was 13.4 (5 to 20 cig/day) in smokers and the mean FTND score was 2.65 (0 to 7). Eight patients had a FTND ≥4 (Table 1). The periodontal characteristics were also similar in both groups. All selected patients suffered from severe periodontitis: 29 patients (10 in smokers, 11 in non-smokers) were classified as chronic periodontitis, 11 patients as aggressive periodontitis. The mean number of missing teeth per patient was 3.55 in smokers and 3.35 in non-smokers. The mean PPD was 4.03 mm in smokers and 3.91 mm in non-smokers. The percentages of sites with PPD > 3 mm and PPD > 7 mm were 54.7 and 3.86 in smokers and 49.9 and 5.14 in non-smokers. The mean follow-up duration was 3.73 years in smokers and 3.03 years in non-smokers. The number of attended visits/year and the percentages of compliance were 1.93 (±0.92) and 65.5% in smokers and 2.16 (±0.61) and 72.1% in non-smokers. Ten smokers and 11 non-smokers received antibiotic treatment. Limited periodontal surgery was only performed on 3 patients in one quadrant (2 smokers and 1 non-smoker) during the whole follow-up period.

**Table 1:** Comparison of baseline and treatment characteristics in smokers and non-smokers

Characteristics	Baseline				Interval of credibility
	Total	Smokers	Non-smokers	Diff	
	(N=40)	(N=20)	(N=20)		
Age (years)	48.2 (12.17)	45.9 (10.58)	50.45 (13.47)	15	(-11.93; 3.51)
Missing teeth (n)	3.45 (4.01)	3.55 (4.57)	3.35 (3.24)	8	(-0.94; 1.38)
Diagnosis CP (n)	29	14	15	5	(-0.31; 0.22)
Cig/day (n)	/	13.4 (4.48)	/	/	
Fagerström score	/	2.65 (1.84)	/	/	
Mean PPD (mm)	3.97 (0.76)	4.03 (0.78)	3.91 (0.77)	1.5	(-0.02; 0.2)
PPD > 3 mm (%)	52.3 (18.1)	54.70 (19.97)	49.91 (16.68)	40%	(1%; 7%)
PPD > 7 mm (%)	4.49 (5.39)	3.86 (4.43)	5.14 (6.29)	15%	(-2%; -1%)
Follow-up years (n)	3.37 (2.29)	3.73 (2.34)	3.03 (2.24)	3	(-0.51; 1.82)
Compliance (%)	68.8 (26.6)	65.5 (31.8)	72.1 (20.4)	40%	(-43%; 7%)
Antibiotics (n)	21	10	11	5	(-0.34; 0.24)

Values are given as means (SD). Diff: estimated allowed difference between smoker and non-smoker groups to be considered clinically similar; cig/day: number of cigarettes per day; CP: chronic periodontitis.

### Change of Periodontal Parameters During Follow-up Depending on Smoking Status

Mean PPD, numbers and percentages of PPD that demonstrated a significant improvement during treatment were evaluated. The mean PPD was reduced by 1 mm, while a 2-fold and a 4-fold reduction of PPD > 3 mm and PPD > 7 mm percentages were observed, respectively. Significant differences were observed for PPD changes between smokers and non-smokers. Mean PPD, PPD > 3 mm and PPD > 7 mm percentage reductions were 1.03, 1.48 and 2.57 less pronounced in smokers than in non-smokers, respectively. The total number of worsening sites (PPD increase > 2 mm) was 2.00 (1.95%) (Table 2). It was significantly higher (3.11) in smokers than in non-smokers. Furthermore, smokers presented a 3-fold higher risk for periodontitis progression corresponding to at least 3 sites on two different teeth with PPD increase > 2 mm (RR=3.33). The mean TL was 0.30 and did not significantly differ between smokers (0.27) and non-smokers (0.33). The multilevel analysis showed that the factors acting at the site-level contributed about 71.6%, 52.7%, and 35.9% to the variation of mean PPD, PPD > 3 mm and > 7 mm percentage reductions, respectively (Table 3).

**Table 2:** Periodontal parameter changes during treatment

Parameters	Mean (SD)		
	Total	Smokers	Non-smokers
∆Mean PPD (mm)	0.90 (0.64)*	0.78 (0.66)	1.01 (0.61)#
∆PPD > 3 mm (%)	22.2 (12.6)*	19.2 (14.0)	25.3(12.6) #
∆PPD > 7 mm (%)	3.40 (5.12)*	2.29 (4.06)	4.62 (5.87) #
PPD increase > 2 mm (n)	2.00 (1.95)	2.70(2.12)	1.30(1.53) #
Progression (n)	13	10	3#
Tooth loss/year (n)	0.30 (0.58)	0.27 (0.56)	0.33 (0.62)

∆: reduction of parameter between baseline and final examination; *significant difference between baseline and final examination pr<0.0001. #Significant difference between smokers and non-smokers pr < 0.01; pr: beta > 0 probability

**Table 3:** Multilevel analysis of periodontal parameter changes during treatment depending on smoking status

Parameters	beta0 (IC)	beta TRT NS/S (IC)	prTRT NS/S	var Patient (%)	var Tooth (%)	var Site (%)	total var
Mean PPD	1.502 (1.226, 1.735)	-0.032 (-0.057, -0.006)	**0.0067**	0.076 (12.67)	0.094 (15.67)	0.429 (71.65)	0.599
%PPD > 3 mm	1.639 (0.3266, 2.7620)	-0.393 (-0.587, -0.198)	**0.0001**	0.793 (22.44)	0.878 (24.82)	1.864 (52.72)	3.536
%PPD > 7 mm	-2.778 (-3.721, -1.806)	-0.945 (-1.425, -0.477)	**0.0000**	1.262 (45.56)	0.513 (18.53)	0.994 (35.89)	2.770
PPD increase > 2 mm	-4.378 (-5.253, -3.528)	-1.137 (-1.878, -0.420)	**0.0011**	0.573 (28.09)	0.897(43.96)	0.570 (27.93)	2.040

TRT NS/S: treatment and smoking effect; pr: beta > 0 probability, var: variance, (IC): interval of credibility (2.5%, 97.5%), (%): percentage of the total variance; significant difference in bold, pr < 0.05 or > 0.95.

### Change of Periodontal Parameters Depending on Arch, Tooth Type and Smoking Status

At the final examination, the reduction of PPD > 7 mm percentage and the number of worsening sites were higher in the maxilla than in mandible for the whole group, while reduction of mean PPD and PPD > 3 mm percentages were similar. The improvement of these parameters except worsening sites was significantly less pronounced in the maxilla of smokers than non-smokers. The reduction of PPD > 7 mm was 6 times higher in the maxilla of non-smokers than in smokers, while this reduction was similar for mandibles. Reductions of mean PPD, PPD > 3 mm and PPD > 7 mm percentages were also significantly lower in molars of smokers. No difference was observed between smokers and non-smokers for non-molars (Table 4). The multilevel analysis showed that the factors acting at the site-level contributed about 95.7%, 53.7%, and 36.3% to the variation of mean PPD, PPD > 3 mm and 7 mm percentage reductions between the maxilla and mandible, and 91.7%, 62.5%, 40.6% between non-molars and molars, respectively (Table 5).

**Table 4:** Periodontal parameter changes during treatment depending on arch and tooth type

	Mean (SD)					
	Total		Smokers		Non-smokers	
	Max	Md	Max	Md	Max	Md
Parameters						
∆Mean PPD (mm)	0.90 (0.61)	0.93 (0.84)	0.69 (0.49)	0.94 (1.01)	1.12 (0.66)#	0.92 (0.66)
∆PPD > 3 mm (%)	23.5 (13.6)	20.9 (17.3)	19.8 (11.7)	18.8 (18.9)	27.2 (14.4)#	23.1 (15.8)
∆PPD > 7 mm (%)	3.71 (6.49)	3.14 (5.0)*	0.98 (0.76)	3.14 (4.46)	6.44 (7.10)#	3.13 (4.31)
PPD increase > 2 mm (n)	1.25 (0.96)	0.75 (1.25)*	1.75 (1.58)	0.95 (1.31)	0.75 (0.96)	0.55 (1.19)
	NM	M	NM	M	NM	M
Parameters						
∆Mean PPD (mm)	0.91 (0.43)	0.82 (0.78)*	0.84 (0.72)	0.52 (0.71)	0.98 (0.65)	1.11 (0.76) #
∆PPD > 3 mm (%)	23.5 (15.3)	18.2 (17.7)	22.1 (17.1)	9.72 (14.7)	25.0 (13.6)	26.7 (16 .6) #
∆PPD > 7 mm (%)	3.20 (4.80)	3.60 (1.00)	2.09 (2.26)	1.62 (2.74)	4.31 (5.17)	5.58 (7.09) #
PPD increase > 2 mm (n)	0.92 (1.65)	1.07 (1.24)*	1.35 (2.05)	1.35 (1.38)	0.50 (1)	0.80 (1.05)

Mx: maxilla, Md: mandible, NM: non-molars, M: molars; ∆: reduction of parameter between baseline and final examination; *Significant difference between maxilla and mandible or non-molars and molars pr<0.05 ; #Significant difference between non-smokers and smokers pr<0.05 or >0.95.

**Table 5:** Multilevel analysis of periodontal parameter changes during treatment depending on smoking status, arch and tooth type

Mx/Md	beta0 (IC)	betaTRT Mx/Md (IC)	pr TRT Mx/Md	betaTRT NS/S Mx/Md (IC)	prTRT NS/S Mx/Md	Var Patient (%)	Var Tooth (%)	Var Site (%)	Total var
mean PPD	-1.094 (-1.765, 0.723)	0.007 (-0.025, 0.039)	0.662	-0.067 (-0.106, -0.027)	**0.000**	0.129 (2.23)	0.118 (2.04)	5.519 (95.71)	5.766
%PPD > 3 mm	1.944 (0.723, 3.167)	0.157 (-0.085, 0.399)	0.899	-0.480 (-0.746, -0.214)	**0.000**	0.789 (22.64)	0.821 (23.56)	1.874 (53.78)	3.484
%PPD > 7 mm	-2.613 (-3.626, -1.495)	-0.433 (-0.954, 0.080)	**0.049**	0.799 (-0.063, 1.681)	**0.965**	1.274 (45.41)	0.511 (18.21)	1.02 (36.36)	2.805
PPD increase > 2 mm	-4.033 (-4.975, -3.108)	-0.769 (-1.720, 0.124)	**0.046**	-0.032 (-1.164, 1.067)	0.4794	0.576 (28.90)	0.857 (42.95)	0.561 (28.14)	1.996
NM/M	beta0 (IC)	betaTRT NM/M (IC)	prTRT NM/M	betaTRT NS/S NM/M (IC)	prTRT NS/S NM/M	Var Patient (%)	Var Tooth (%)	Var Site (%)	Total var
Mean PPD	-0.362 (-1.940, 0.591)	0.102 (0.068, 0.137)	1	-0.070 (-0.112, -0.027)	**0.000**	0.122 (4.17)	0.119 (4.07)	2.682 (91.75)	2.923
%PPD > 3 mm	1.280 (0.181, 2.396)	-0.004 (-6.166, 6.189)	0.499	0.162 (0.012, 0.312)	**0.983**	0.797 (26.99)	0.309 (10.46)	1.846 (62.53)	2.952
%PPD> 7 mm	-3.040 (-4.030, -2.066)	0.016 (-6.229, 6.243)	0.503	0.824 (0.470, 1.204)	**1.000**	1.259 (51.72)	0.187 (7.68)	0.988 (40.59)	2.434
PPD increase > 2 mm	-4.741 (-5.639, -3.859)	1.259 (0.443, 2.104)	**0.9984**	0.453 (-0.618, 1.561)	0.7928	0.580 (34.27)	0.542 (32.03)	0.570 (33.68)	1.694

TRT Mx/Md: comparison of treatment effect between maxilla and mandible; TRT NM/M: comparison of treatment effect between non-molars and molars; TRT Mx/Md NS/S: comparison of treatment and smoking effect between maxilla and mandible; TRT NM/M NS/S: comparison of treatment and smoking effect between non-molars and molars; pr: beta > 0 probability, var: variance, (IC): interval of credibility (2.5%, 97.5%), var: variance, (%): percentage of the total variance; significant difference pr < 0.05 or > 0.95 in bold.

### Demographic, Periodontal and Treatment Characteristics, Smoking, Addiction and changes of Periodontal Parameters

Multivariate and multilevel regression analysis showed that few demographic, periodontal and treatment characteristics other than smoking, arch and tooth type influenced periodontal parameter changes in the whole group, as well as in smokers and non-smokers. Mean PPD reduction diminished with age (pr=1). The number of cigarettes and smoker classification as light (5 to 10 cig/day) vs moderate (11 to 20 cig/day) did not independently influence periodontal outcomes (data not shown). However, patients with a FTND > 3 demonstrated a higher TL (0.66 vs 0.02).

## Discussion

Our study showed that the efficacy of the whole periodontal treatment including APT and SPT was selectively impaired in moderate smokers suffering from severe periodontitis. This reinforced the hypothesis that smoking has a specific deleterious effect on periodontal tissue healing.^31,41^ Few studies have investigated the effect of smoking effect both APT and SPT.^9,20,30,43^ However, periodontal response to treatment is a continuous process and the short-term effect term could change (worsen/improve) over the long-term, emphasising the clinical relevance of considering periodontal treatment as a whole.^9,30,63^ The changes of various periodontal parameters were significantly less favourable in smokers than in non-smokers, especially for moderate and deep periodontal pockets. However, the observed difference of mean PPD changes between smokers and non-smoker (0.23 mm) appeared statistically and clinically less relevant using multilevel analysis. Similarly slight or no significant differences between smokers and non-smokers^9,14,30,30,63^ and no difference^45,49^ have been observed in the short and long term, suggesting that mean PPD reduction is not the best parameter to determine the effect of smoking on periodontal treatment also in the long term.

The multilevel analysis showed that the site-level factor contributed largely (around 70%) to the total variance of mean PPD changes, as previously observed with other multilevel methods for lingual vs buccal site localisation, plaque accumulation and PPD at baseline.^14,63^ These data suggest that moderate to deep pocket changes may be more influenced by factors acting at the patient-level, such as smoking. In the study by Wan et al,^63^ the reduction of PPD > 4 mm percentage was 1.6-fold higher in non-smokers than in smokers. In our study, the percentage reduction of diseased sites (PPD > 3 mm) was 1.48-fold higher in non-smokers compared to smokers. This negative effect of smoking was amplified in deep pockets, with a 2.57-fold higher reduction in non-smokers for PPD > 7 mm. The same ranges of amplification have already been described for the probability of pocket closure in the short term^59^ and can apparently persist, as shown here.

Interestingly, periodontal disease progression appeared to be significantly increased in smokers, as previously described.^37^ The numbers/percentages of sites with PPD ≥ 5 mm,^5^ with no decrease of PPD after APT at 10 weeks^27^ and PPD deterioration > 2 mm,^19^ and CAL ≥ 3 mm^37,42^ during SPT, were correlated with smoking. However, in the study by Fisher et al,^19^ the recurrent sites with an increase of PPD were not related to smoking. The lower percentage of recurrent sites (0.85%) may explain this contradictory result. Furthermore, in our study, 50% of smokers (15% of non-smokers) presented disease progression, corresponding to 77% of patients with periodontitis progression in the whole group. The negative impact of smoking during SPT on the number of patients with periodontitis progression has been previously shown in various studies.^5,37,42^ Our data extended this notion to the whole range of periodontal treatment, including APT and SPT.

The negative effect of smoking on periodontal treatment outcomes also appeared to be influenced by anatomical factors. The improvement of periodontal status was significantly more pronounced in the maxilla of non-smokers than smokers. This influence of arch on treatment response for smokers has not been frequently described. It has been observed in studies with tooth- and site-level analysis for residual PPD ≥ 5 mm + BOP,^9^ and molar loss,^50^ but was not observed for mean PPD reduction.^63^ The periodontal status of maxillary teeth has been shown to be constantly worse in smokers than in non-smokers,^29^ suggesting the existence of a local effect in addition to a systemic effect of smoking on periodontitis severity.^9,23^ Similarly, our data showed that molars did not respond well to periodontal treatment compared to non-molars. This effect was amplified in smokers. A similar trend has been previously described for pocket closure (PPD ≥ 4 mm) in the short term,^59^ as well as for tooth loss.^36^ However, a tooth/site-dependent increase of residual PPD ≥ 5 mm + BOP has been described in maxillary single-rooted teeth of smokers but not in maxillary multi-rooted teeth.^9^ The concomitantly observed decrease of BOP% and increase number of PPD ≥ 6 mm during 5-year SPT in smokers suggested that the association of these two clinical signs at the site level in smokers may not reflect the same periodontal treatment morbidity as in non-smokers.^46^ These data pointed out the importance of considering the severity of molar periodontal destruction in smokers before starting periodontal treatment.^59^

Concerning other potential influencing factors on the observed periodontal treatment outcomes, initial periodontal disease severity and percentages of deep pockets were similar between smokers and non-smokers, while in other studies, the initial severity of periodontitis was frequently higher in smokers.^9,46^ The number of patients suffering from severe chronic or aggressive periodontitis was also similar in smokers and non-smokers, anticipating a potentially different response between these two types of periodontitis.^16^ Treatment modalities, such as antibiotic use and compliance, were also equivalent in both groups. The multivariate multilevel regression analysis did not reveal selective influences of initial periodontal diagnosis and treatment modalities, i.e. antibiotic use, follow-up duration and compliance, confirming that smoking independently and predominantly influenced periodontal treatment outcomes in the mid-term in our study population. These data were in agreement with the lack of a different influence of the type of severe periodontitis (chronic/aggressive) and antibiotic treatments in smokers vs non-smokers.^11,16^ A dose-effect of smoking was not observed in our study, contrary to previous data on periodontal treatment outcomes.^36,37^ In our study, smoking effect evaluation was based on patient self-reports and not on objective measurements of nicotine level or exhaled carbon monoxide, which may also limit the interpretation of results.^9,31^ However, this discrepancy could be due to the absence of heavy smokers (> 20 cig/day) and to the relatively small size of the smoker group.^1^ The absence of heavy smokers could reflect the observed reduction of number of cigarettes consumed in France in the last decade.^58^ In our study, TL was not influenced by smoking status, as previously shown in some studies.^37^ However, smokers with a FTND ≥ 4 corresponding to the optimum cut-off score of FTND to establish nicotine dependence^26^ showed a higher TL rate. A low FTND has been shown to be a predictor of smoking cessation,^35^ also in periodontally treated patients.^28^ However, FTND, especially the time of the first cigarette after waking up, was also independently associated with upper-respiratory and digestive-tract cancers regardless of smoking intensity and duration.^32^ These data suggested that evaluation of nicotine dependence via FTND could capture additional negative effects of smoking on periodontal treatment outcomes, which merit further investigations.

## Conclusion

Within the limitations of this study, our data demonstrated a negative effect of smoking on specific periodontal treatment outcomes in the mid-term, in light of moderate smokers suffering from severe periodontitis. The failure of periodontal treatment associated with PPD/at-risk tooth site and periodontitis progression were selectively amplified in smokers. Our data could allow providing patients with more specific information their individual smoking risk. For patients who did not quit smoking, tailored APT and SPT based on periodontitis severity and addiction evaluation may also be considered in individual risk. Complementary anti-infectious treatments and reinforcement of patient compliance may be necessary.^7,47,57^ Finally, a tool such as FTND should be used rather than number of cigarettes/day only to precisely determine the addiction level and characterise smoking habits. Such analyses will be useful in adapting smoking addiction treatment strategies.
